# Longitudinal mentorship to support the development of medical students’ future professional role: a qualitative study

**DOI:** 10.1186/s12909-015-0383-5

**Published:** 2015-06-03

**Authors:** Susanne Kalén, Sari Ponzer, Astrid Seeberger, Anna Kiessling, Charlotte Silén

**Affiliations:** 1 Department of Clinical Science and Education, Karolinska Institutet, Södersjukhuset, SE-118 83 Stockholm, Sweden; 2 Department of Clinical Science, Intervention and Technology, Karolinska Institutet, Stockholm, Sweden; 3 Department of Clinical Sciences, Danderyd Hospital, Karolinska Institutet, Stockholm, Sweden; 4 Department of Learning, Informatics, Management and Ethics, Karolinska Institutet, Stockholm, Sweden

**Keywords:** Mentorship, Undergraduate medical students, Professional competence, Professional development

## Abstract

**Background:**

Mentoring has been employed in medical education in recent years, but there is extensive variation in the published literature concerning the goals of mentoring and the role of the mentor. Therefore, there is still a need for a deeper understanding of the meaning of mentoring for medical students’ learning and development. The aim of this qualitative study is to explore how formal and longitudinal mentoring can contribute to medical students’ professional development.

**Methods:**

Sixteen medical students at a Swedish university were interviewed individually about their experiences of combined group and one-to-one mentoring that is given throughout their studies. The mentoring programme was focused on the non-medical skills of the profession and used CanMEDS roles of a physician for students’ self-assessment. Data were analysed using a latent, interpretive approach to content analysis.

**Results:**

The results comprise three themes: *Integrating oneself with one’s future role as a physician, Experiencing clinical reality with the mentor creates incentives to learn* and *Towards understanding the professional competence of a physician.* The mentorship enabled the students to create a view of their future professional role and to integrate it with their own personalities. The students’ understanding of professional competence and behaviour evolved during the mentorship and they made advances towards understanding the wholeness of the profession. This approach to mentorship supported different components of the students’ professional development; the themes *Integrating oneself with one’s future role* and *Towards understanding the professional competence of a physician* can be regarded as two parallel processes, while the third theme, *Experiencing clinical reality with the mentor creates incentives to learn,* promotes these processes.

**Conclusions:**

Formalized and longitudinal mentoring focusing on the non-medical skills can be recommended to help medical students to integrate their professional role with themselves as individuals and promote understanding of professional competence in the process of becoming a physician.

## Background

Mentoring programmes of different designs have been used in medical education in recent years [1–13]. There is extensive variation in the medical education literature concerning the goals of mentoring and the role of the mentor, and it has been suggested that further research on the efficiency and meaning of mentoring is needed [1, 5, 14]. To contribute to the understanding of how mentoring can be used to support medical students’ professional development, we have explored this area in a Swedish context using a qualitative research approach [15–17].

In general, a mentoring relationship is embedded within a career context and provides two functions for mentees: a career function and a psychosocial function [18, 19]. The function with a focus on career development is more to the fore in North America and the function involving psychosocial support is more to the fore in Europe [20]. It is important to view mentoring in the specific context where it occurs since the benefits from mentoring are context-dependent [2, 20], which seems reasonable since there can be different perceptions of mentoring in different parts of the world. The understanding of mentoring in this study is in line with a description developed by the Standing Committee on Postgraduate Medical and Dental Education, SCOPME, in the UK: …the process whereby an experienced, highly regarded, empathic person (the mentor) guides another individual (the mentee) in the development and re-examination of their own ideas, learning, and personal and professional development. The mentor, who often, but not necessarily, works in the same organization or field as the mentee, achieves this by listening and talking in confidence to the mentee ([21], p.1).

In undergraduate medical education most research on mentoring is reported from North America [1, 2], for which reason research in this field from other geographic areas has been called for [2]. A review [1] of the PubMed literature published during 2000 to 2008 on mentoring aimed at supporting medical students’ professional and personal development included 25 studies from North America. The authors state that mentoring would be appreciated more if its effects were more clearly documented [1]. A study from Germany showed that 22 of 36 medical schools offered mentoring programmes for their students, but most of the programmes were not evaluated or published, for which reason the authors stated that controlled studies are needed to compare the efficiency of different forms of mentoring [22]. Efforts have been made to develop instruments to measure the effectiveness of mentoring, but such instruments tend to limit quantification of results related to a person-, relationship-and programme-specific context [6]. Frei et al. [1] argue that it is important to capture the individuals’ own experiences by means of qualitative methods that explore the area of mentorship involving interactions and relationships between individuals.

Professional development can be seen from different perspectives. In this study, we have chosen to relate it to the perspective of professional competence. Professional competences of a physician have been defined in more recent years with new recommendations for educators [23–25], and competence-based education with measurable outcomes is now widely implemented [26]. Beyond competence in medical science and clinical skills, competences related to non-medical skills are emphasized. Epstein & Hundert [25] described four functions included in professional competence: cognitive function, integrative function, relational function and affective/moral function. The Royal College of Physicians and Surgeons of Canada developed the CanMEDS 2005 Framework and connected the physician’s competences to seven roles: medical expert, communicator, collaborator, manager, health advocate, scholar and professional [24]. A description of professional competence by Forslund [27, 28] illustrates how different components in professional competence relate to each other and create wholeness with the individual behind the profession. This model includes six components: knowing the goals of the profession, knowing the ethical norms, having a systematic theoretical base, acquiring a set of methods, the personal profile and evaluation of one’s work. The personal profile in this model is created as a combination of the individual’s personality and previous knowledge and the ethical codes, theories and methods of the profession. Forslund states that the personal profile is close to professional identity and constitutes the base for every professional action [27, 28]. How mentoring focusing on the non-medical aspects of the profession can support medical students’ learning and development is therefore of great interest. To explore this area, it is important to capture the students’ own experiences by means of qualitative research methods. The aim of this study was to explore how formal longitudinal mentoring can contribute to medical students’ professional development.

## Methods

This study took place at Karolinska Institutet, Sweden, where, since 2007, a mandatory combined group and one-to-one mentoring programme has been running throughout the medical education programme. The medical education programme at Karolinska Institutet comprises 5.5 years and consists of four terms with mainly basic science courses, followed by seven terms with mainly clinical courses. One year comprises two terms in Sweden. The goal of the mentoring programme is to facilitate students’ professional and personal development. Groups of four students meet their mentor on a ‘workshop day’ once a term, terms 1 to 11. The mentoring programme consists of three activities focusing on the physician’s professional role: (1) Each student reflects on and discusses his/her own development individually with the mentor, using a self-assessment form based on the CanMEDS framework for the physician’s professional roles and competences [24]. Students, acting individually with the mentor, set up goals and perform an action plan for improvement in these roles. (2) The group watches videos focusing on psychological and ethical aspects of the interaction between the physician and patient, with increasing complexity with each term. This is followed by a discussion with peers and the mentor about how to handle such situations professionally. (3) The mentoring programme also offers opportunities for the students to visit the mentor in connection with their clinical work.

The mentors are physicians working actively in the health care system. Some of them are also clinical teachers or supervisors. They are recruited as volunteers based on interests and recommendations. The role of the mentor is in line with SCOPME’s description of mentoring [21], i.e., to support the students’ professional and personal development in confidence, and not to assess or judge their performance. The student groups and mentors are randomly matched. Before every workshop day the mentors are invited by the faculty to meetings to prepare for the video sessions and to share experiences with other mentors.

**Fig. 1:**
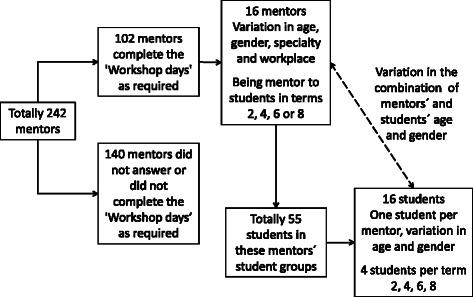
Flowchart of the sampling process

For data collection, individual semi-structured interviews were used to capture the students’ experiences [29, 30] and a maximum variation sample process [30, 31] was used to secure a wide variety of participants and breadth in the data. The selection of students started with the selection of their mentors (Fig. 1). One hundred and two mentors (out of 242) declared in an electronic survey that they had completed all of the three activities in the mentoring programme, which was the criterion for their students to be eligible for the study. A strategic sample of 16 mentors was selected with a wide variation in age, gender, specialty and workplace. Finally, 16 students in these mentors’ student groups were strategically selected with variation in age and gender, one student per mentor, four students from each of the terms 2, 4, 6 and 8. Two of the initially selected students declined to participate and we were not able to contact four by telephone or e-mail, so new participants were selected following the same sampling procedure. The included students were in the age range 20–29 years and consisted of eight men and eight women.

The 16 individual interviews were conducted by one of the authors (SK) who had an external role to the medical education programme at Karolinska Institutet. The interviews yielded 13 h of recorded material. The material covered the students’ experiences of the different parts of the workshop days, their development and their mentorship experiences. The students were informed about the study in an e-mail letter signed by the researchers primarily responsible for the study and not involved in the medical education programme (SK, SP, CS) and were thereafter asked by telephone (SK) if they were willing to participate. It was optional for the students to participate and they were informed that they could withdraw whenever they wanted to with no impact on their course marks. Their informed consent was obtained and they were guaranteed full confidentiality. This study was conducted according to the Helsinki Declaration and was approved by the Regional Ethical Review Board at Karolinska Institutet in Stockholm.

An interpretive and latent approach to content analysis was used to extend the existing knowledge in this area [32, 33]. The analysis was conducted manually by SK, CS and SP. The recorded interviews were transcribed verbatim and yielded two domains of data: one relating to the type of space for development the mentorship created [17] and one relating to the students’ professional development, the latter creating the dataset for this study. Meaning units were identified, condensed and then entered into the NVivo 9 software package for qualitative data analysis [34], where they were labelled with a code close to the content. Codes related to each other were organized into categories and finally interpreted as three main themes (Table 1). Codes and categories were derived from the manifest content, while the themes were the result of an interpretation of the meaning of the latent content. All steps in the analytical process were performed manually. The NVivo 9 software package was only used as a tool to organize and handle the data digitally. Research triangulation and frequent debriefing sessions were used throughout the process. The researchers moved back and forward between the different steps and codes, categories and themes were compared and discussed from different perspectives in the research group until a consensus was reached. Three of the authors (SK, SP, CS) conducted the main part of the study. Two of the authors (AS, AK) involved in the mentoring programme contributed to the understanding of the context and the relevance of the result. They joined the process in the last stages to ensure that prior assumptions and potential student-staff relationships would not impact the data collection and analysis.

**Table 1:** Scheme of codes, categories and themes

Code	Category	Theme
Difficult to see the future goal	A vision of the future as a physician	Integrating oneself with one’s future role as a physician
Seeing the future goal through experience in the clinic		
Insight into the profession		
See that you have made the right choice of profession		
Having no physician in the family or among friends		
How to combine professional life with private life	Hope to manage it	
It will work		
Fellowship		
Self-awareness of one’s own personality	The personal and the professional are interlinked	
Advice for study tactics versus other interests		
Link the professional with oneself-student		
Link the professional with oneself–mentor		
Distancing oneself		
Clinic spurs motivation	Clinical experiences with the mentor spur motivation	Experiencing clinical reality with the mentor creates incentives to learn
Looking forward to work as a doctor		
Positive feelings from experiencing reality		
Excitement and fun in the clinic–different terms		
Desire for exciting experiences		
Being invited		
Clinic spurs motivation to study and learn	Learning about medicine in the clinic	
Connect theoretical knowledge to practice		
Novice in the clinic		
Interest in medicine		
Using the self-assessment form	Orientation concerning the physician’s areas of competence	Towards understanding the professional competence of a physician
Self-assessment areas		
Self-assessment difficult and diffuse		
Sets goals to reach		
Behaviour in the professional role	Learning about professional behaviour	
Learning about patient encounters		
Getting perspectives		
Preparation for difficult situations		
Becoming more self-confident		
Collegial interaction		

## Results

The interpretation of the manifest content resulted in three main themes: *Integrating oneself with one’s future role as a physician, Experiencing clinical reality with the mentor creates incentives to learn* and *Towards understanding the professional competence of a physician.* The themes are presented in the following based on their categories, along with illustrative quotes. Themes, categories and codes are presented in Table 1.

### Integrating oneself with one’s future role as a physician

This theme was interpreted on the basis of three categories: a *vision of the future as a physician, hope to manage it* and *combine the professional role with oneself as a person*.

The students saw their professional life as a physician as being far and distant in the future. Clinical experiences with the mentor created a view of how their future life will be. They got an early glimpse of the profession by talking to the mentor who was a physician, following him/her in the clinic, seeing the daily tasks of a physician and how the health care system is organized. They got a picture of the profession by seeing *what* the mentor does and *how* the mentor does it–both positive and negative sides of the coin.

…well, I think it is important for me to…to know better what I can expect…and this feeling, for the profession, well it feels very diffuse. It is there somewhere, in the future. (female, term 4)

It brought insight into the profession when the mentor, in a relaxed atmosphere, shared his/her experiences concerning how to handle different situations. The students’ understanding of the profession became clearer, especially if they did not know any physician in person before. They could confirm that they were in the right place and had chosen the right educational programme.

…in the preclinical terms, when you felt, ‘Oh, this is very hard. Is this really what I want to do?’… But when I followed her in her work, it was really like ‘Oh, I am in the right place.’ (female, term 8)

The students talked with the mentors about how to combine professional life with private life, having a family and doing research, and how to behave as a physician in private life. To see how the mentor did and how he/she handled the situation gave hope of managing it oneself one day. This involved private life, the professional role and different career choices. The mentor had gone through what the students had in front of them. During the mentorship, when reflecting on the role and the competences of a physician, the students became aware of their own personality, behaviour and characteristics, how they interact with others and what they needed to develop and train further to cope with the education and the profession. They imagined themselves as physicians and how they would fit into the role. It was good to verbalize and put personality and development into words and they noticed personal development and their own maturation during the ongoing programme.

…I was very much like this…wanted to take care of everything myself and to have control and… ‘Yes, just ask me, I’m a living timetable’ type. But then I felt that no, I don’t think this is fun anymore. So that part I have actually changed. (female, term 4)

The students could see the mentor shifting from being the professional person into the person behind the professional role, both sides were there, but they shifted from situation to situation depending on which side was to the fore. They realized that the professional role has to be integrated with oneself as a person, that you cannot separate the profession from the person.

… just to see her both when she is working and when she is not working, when she is more like…anyone else, almost. Ahem…and to integrate that picture, well, that, yes, I will be able to be both a human being and a physician. (female, term 4)

They came to the insight that the personal and the professional are associated with each other, and to find a balance in this combination you have to be a professional based on the person you are.

Well…when talking about the professional role, then I think personal development comes into that picture, they merge in a way… So there is a very blurred boundary between them…for me, it has been a personal development. And I think the personality is part of the professional in the end, so that is very important. (female, term 8)

### Experiencing clinical reality with the mentor creates incentives to learn

This theme was interpreted on the basis of two categories: *Clinical experiences with the mentor spur motivation* and *Learning about medicine in the clinic*.

To follow the mentor in the clinic and get clinical experiences gave rise to motivation and encouragement. The students got a view of the reality at a hospital and looked forward to working as a physician. It was a positive experience to be in the clinical environment and see what happens there, to get a first contact with real life at a hospital. It increased their motivation to study during difficult preclinical courses. They expressed their experiences with the mentor in the clinic as being encouraging, engaging, interesting and exciting.

…that’s the strongest experience so far in the training…which I got now: to really be there, and actually stand there pressed against the wall and be scared to death to breathe too loudly and disturb anyone; such things really impress… I got a view of reality, I became more motivated by that, I’ve been studying better since then, because I have another motivation. One knows that…yes, this, just this, is what I need to know because I will face such situations where these things happen. (male, term 2)

The mentors invited the students to follow them in the clinic whenever they wanted to. The students felt welcome and also had the opportunity to suggest interesting activities they wanted to see or practice. The early access to the clinic with the mentor motivated and inspired them to theoretical studies because they saw what they needed the knowledge for; it brought meaning to their theoretical studies.

…the first two years are very much basic science, and there is so very much theory, and you just have to sit there and learn about cells and reactions and…ion pumps and all that stuff. So it was nice to come here and see and, yes, meet the patient, and you actually apply all of this in the clinic. (male, term 6)

They could put the theory into a clinical context and use what they had read in the books in a clinical reality, which created meaning for the academic world. When they were in the clinic with the mentor they asked questions about medical issues, and they could reveal their ignorance to the mentor without the risk of being judged. The students did not participate actively in situations with the patients but acted more like observers. They saw patients with symptoms and diseases they had not encountered before, and saw that reality is not always consistent with what is in the books. It was most exciting to follow the mentor in the clinic in the early terms. In later terms, when they had come further in their education and had more clinical experience, it was less interesting, depending on the mentor’s workplace. In later terms it was more interesting and important to discuss one’s own experiences from clinical courses with the mentor. The students appreciated being invited to the clinic, but thought they could have taken better advantage of the invitation.

### Towards understanding professional competences of a physician

This theme was interpreted on the basis of two categories: *Orientation about the physician’s areas of competence* and *Learning about professional behaviour*.

The students thought it was hard to assess themselves according to the physician’s professional roles in the self-assessment form, especially in the early terms. It was hard to understand the meaning of the different areas of competence. In the first step, it was easier to focus on strengths and weaknesses connected to their own personal characteristics.

Yes, the assessment form, the areas about myself, somehow, there you know from other situations how you function. Somehow, maybe the clearest is leadership, which I think I will be good at, without knowing how I will function in the medical context, because I have done much of that in other contexts. (male, term 2)

The students tried to imagine how they would react in different situations where they sometimes felt constrained. Students in the early terms understood the role of the medical expert best, even if they felt absolutely ignorant in that area. The role of the health advocate was most diffuse and hard to describe and understand, and several roles were perceived as irrelevant.

… from the very beginning, many of these competence assessments were… totally… I had no idea about what they even… I had no connection to them, I didn’t feel they were relevant. That is, early on, when you have just stepped into the clinic. But the longer you are in the clinic, the more relevant they become and you understand what it is all about. (male, term 8)

The roles of Communicator, Collaborator, Manager, Scholar and Professional were mentioned by students in later terms and these roles acquired more meaning the farther they came in their education and they could relate to something concrete from their own experiences. They also mentioned leadership, organizing and planning, scientific ability, professional development and empathy as competences of a physician. The different parts of the profession became clearer with time and they got a sense of the wholeness and the complexity of the profession.

The students became aware of the importance of physicians’ behaviour and paid attention to attitudes to patients, both in the videos and by watching how the mentor acted in clinical situations, as well as by reflecting on their own experiences. Students in the early terms had practically no experience of this and talked about templates and strategies to train and asked the mentors for practical tips about how to handle different situations.

…it is quite interesting to follow him how… So it’s a little like what kind of strategies he uses and, in general, attitudes to patients, things like that. (male, term 2)

At the beginning, they were not aware of the fact that difficult patient encounters were a part of the profession, but later on they realized that they will have to face such difficulties in the future and it became important to learn how to interact with patients and prepare for that.

The students discussed and reflected on professional behaviour but thought this was hard to grasp before acquiring their own experience from practice. They tried to find out how they should behave and act in difficult situations, both as physicians and in their private lives. They had both good and bad role models for how they wanted or did not want to become themselves. The videos and the experienced reality provided different examples from which they could find out their own role and style to test and train and gain experience. They discussed ethical situations and felt that professional behaviour and learning to communicate with patients became more and more relevant and important the closer they came to professional life. They realized that they spoke in another way now and had got a platform for future situations.

… in the first term… you knew nothing. And I assumed that, yes, I’m normally ‘socially talented’ and can talk to all people. But then I suddenly realized that now… now I talk in a completely different way. (male, term 6)

The group discussions provided a wider perspective so they could see several ways to handle a certain situation. They noted that it was not always clear what to do and that some decisions are context-dependent with no rights or wrongs. They appreciated being able to reflect and think in these ways before they had to face it all in reality. They also realized that this kind of competence cannot be learned in advance: it has to be learned from one’s own experience. Nevertheless, they got a foundation to rely on and felt better prepared for difficult situations in the future.

… you’ve got a bank of knowledge concerning how to behave in those kinds of situations… So I think you bring with you an attitude and you begin to practice such kinds of thinking for such situations… It is pretty good to have some kind of foundation to rely on when you… because some day will be the first day when you have to face it, to more or less do it on your own. (male, term 8)

Questions of an existential nature stirred up thoughts and were considered to be important to discuss. They partook of the mentors’ experiences of behaviour in difficult situations with both patients and staff, which developed their understanding of professional behaviour. Their attitude to communication skills and learning how to meet patients in different situations changed so as to be seen as a gradual process, and they realized that such a development takes time. They became more aware of their own behaviour after these discussions and said that they had acquired more self-confidence in such situations.

## Discussion

The findings in this study indicate that a longitudinal mentorship programme can help medical students to integrate themselves as individuals with their future role as physicians.

**Fig. 2:**
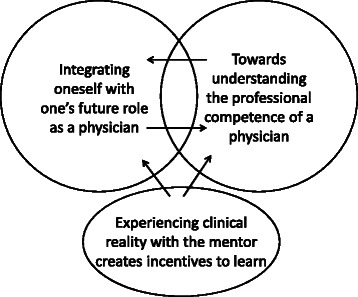
Illustration of the themes, *Integrating oneself with one’s future role as a physician* and *Towards understanding the professional competence of a physician,* as two parallel developmental processes and of the theme, *Experiencing clinical reality with the mentor creates incentives to learn,* as promoting the aforementioned processes

Early clinical experience with the mentor was an incentive; it helped them to imagine their future life as a physician and gave meaning to their theoretical studies. The students’ understanding of professional competence and behaviour evolved during the mentorship and they progressed towards understanding the wholeness of the profession. The themes, *Integrating oneself with one’s future role* and *Towards understanding professional competence*, can be regarded as two parallel developmental processes, while the theme, *Experiencing clinical reality with the mentor creates incentives to learn,* promotes these processes (Fig. 2). The findings in this study suggest that mentoring focusing on the non-medical skills can help medical students to integrate the professional role with their own personalities and promote their understanding of the wholeness of professional competence in the process of becoming a physician.

It has been argued that medical education tends to focus more on learning about the physician’s *doings* rather than *being* and a broader focus on the relationship between the development of competences and the formation of a professional identity has been called for. It has also been pointed out that competences, roles and emergent identities complement one another in the process of becoming a physician [26]. We believe that becoming a physician includes both learning professional competences and forming one’s own professional identity and that development is a change derived from learning. We agree with Goldie who stated, ‘Education in its broadest sense is about transformation of the self into new ways of thinking and relating.’ ([35], p. 641)

This study revealed that learning occurred on different levels. The students’ statements concerning their experience of the mentorship indicated learning and understanding, and also motivation and the creation of meaning. We relate these findings to understanding and meaningful learning according to Marton & Booth and Mayer [36, 37]. They described learning and understanding in terms of learning and awareness [36] and rote versus meaningful learning [37]. The students’ understanding of the professional competences of a physician was expressed by interpreting and exemplifying different roles, which became easier in the later terms when they could relate to their own experiences. Even if they did not come to an understanding of all the roles, they got a view of the wholeness and complexity of the profession. Our data imply that the students’ understanding of professional behaviour reached a deeper level of learning since they were able to interpret, evaluate and compare different behaviours and situations and stated that they had widened their perspectives. They described professional behaviour as being context-dependent, with no right or wrong, and realized that this cannot be learned in advance; it takes time and has to be learned from experience. A surface approach to learning in this area was seen in the early terms when the students were searching for templates and strategies to train for different patient encounter situations. Students in the later terms indicated an increased understanding of professional behaviour as they noticed that they had changed as individuals during the mentorship and stated that they had a foundation to build on for future situations. The mentorship also contributed to motivation, especially in the early terms when the students were enthusiastic and excited to get legitimate access to the clinical environment with the mentor. They created a view of their future life, which also increased their motivation to study.

In Forslund’s model of professional competence [27, 28], individuals’ personalities are integrated in the ‘personal profile,’ which is the foundation for all professional activities. In this mentoring programme the personal relationship with the mentor helped students to see the profession as being integrated with the person behind the professional role, and the early access to the clinical environment together with the mentor enabled an early start of their own integration into the profession. The recurring self-assessment contributed to awareness of their own personal characteristics and habits, and to evaluating them in relation to the profession of a physician. The students noticed changes in their own behaviour during the mentorship, which might be related to mentoring with a focus on non-medical skills and the professional behaviour of a physician. This model of mentoring leads to reflection on one’s own personality, which can contribute to the students’ development of the professional role and their ‘personal profile’ [27, 28]. This model of mentorship, including a permissive atmosphere and space to develop alongside the educational programme, a personal relationship with a physician and a directed content, enabled the students to integrate the professional with the personal and the private with the whole. The longitudinal mentoring programme enabled the students to start this process at an early stage of their education.

The above results should be regarded as an interpretation based on experiences of the individuals participating in the study. The strength of the study is the comprehensive and methodical sampling procedure, which made it possible to obtain maximal variation in the data. One limitation is that experiences from students in terms 9–11 could not be included because the mentoring programme had lasted for only four years at the time of data collection. Frequent debriefing sessions and investigator triangulation, viewing data from different perspectives with conscious reflexivity, enhanced the credibility and trustworthiness of the results [30, 32, 38]. Efforts were made to provide rich descriptions of the work and relate the findings to existing theories so as to enable transferability of the results to other similar contexts [38].

## Conclusions

This study contributes to a deeper understanding of how longitudinal and formalized mentoring can support medical students’ understanding of the wholeness of the professional competence of a physician and to their integrating themselves as individuals with their future professional role. The study focused on the students’ experiences of mentorship and the enhancement of their professional development. During the mentorship, the students constructed meaning and identities for themselves complementary to their clinical learning experiences. These findings are valuable for medical educators who seek to use a mentoring approach to enhance students’ learning and professional development. Based on our research, we can recommend educators to use longitudinal mentoring, focusing on the non-medical aspects of the profession. Important factors for creating such a developmental environment in a mentoring programme are the neutral role of the mentor, an early and directed focus on the non-medical aspects of the professional role and continuity throughout the educational programme. Future studies are needed to get a more in-depth understanding of how medical students’ professional development occurs over time.

## Abbreviation

SCOPME: Standing committee on postgraduate medical and dental education
